# Uncovering the relation between clinical reasoning and diagnostic accuracy – An analysis of learner's clinical reasoning processes in virtual patients

**DOI:** 10.1371/journal.pone.0204900

**Published:** 2018-10-04

**Authors:** Inga Hege, Andrzej A. Kononowicz, Jan Kiesewetter, Lynn Foster-Johnson

**Affiliations:** 1 Institute for Medical Education, University Hospital of LMU Munich, Munich, Germany; 2 Medical School, University of Augsburg, Augsburg, Germany; 3 Department of Bioinformatics and Telemedicine, Jagiellonian University Medical College, Krakow, Poland; 4 Department of Community & Family Medicine at the Geisel School of Medicine at Dartmouth, Hanover, New Hampshire, United States of America; University of Ulm, GERMANY

## Abstract

**Background:**

Clinical reasoning is an important topic in healthcare training, assessment, and research. Virtual patients (VPs) are a safe environment to teach, assess and perform research on clinical reasoning and diagnostic accuracy. Our aim was to explore the details of the clinical reasoning process and diagnostic accuracy of undergraduate medical students when working with VPs using a concept mapping tool.

**Methods:**

Over seven months we provided access to 67 German and 30 English VPs combined with a concept mapping tool to visualize and measure the clinical reasoning process of identifying problems, differential diagnoses, recommended tests and treatment options, and composing a summary statement about a VP. A final diagnosis had to be submitted by the learners in order to conclude the VP scenario. Learners were allowed multiple attempts or could request the correct diagnosis from the system.

**Results:**

We analyzed 1,393 completed concept maps from 317 learners. We found significant differences between maps with a correct final diagnosis on one or multiple attempts and maps in which learners gave up and requested the solution from the system. These maps had lower scores, fewer summary statements, and fewer problems, differential diagnoses, tests, and treatments.

**Conclusions:**

The different use patterns and scores between learners who had the correct final diagnosis on one or multiple attempts and those who gave up, indicate that diagnostic accuracy in the form of a correct final diagnosis on the first attempt has to be reconsidered as a sole indicator for clinical reasoning competency. For the training, assessment, and research of clinical reasoning we suggest focusing more on the details of the process to reach a correct diagnosis, rather than whether it was made in the first attempt.

## Introduction

Clinical reasoning teaching and assessment is a major aspect in both, healthcare education and research. Healthcare students have to acquire this important skill during their education and continue to further develop it in the workplace. The complex clinical reasoning process includes the application of knowledge to synthesize and prioritize information from various sources and to develop a diagnosis and management plan for a patient. Various models and theoretical frameworks for clinical reasoning have been developed, including a complex model by Charlin et al.[1] or a more teacher-oriented model by Eva [2]. But, despite being a heavily researched topic, it is still not clear, how clinical reasoning is learned and how it can be effectively taught or assessed [3]. Thus, a typical indicator to measure clinical reasoning skills is diagnostic accuracy, which is often defined and assessed as reaching the correct final diagnosis in a first attempt [4].

Web-based virtual patients (VPs) are widely used to train students and healthcare professionals in clinical reasoning [5,6]. VPs provide a safe environment in which learners can develop their clinical reasoning skills at their own pace and learn from diagnostic errors without harming a patient [7]. VPs are typically designed to unfold in a step-by step manner, revealing the information about a patient in a "serial-cue" format. However, evidence about the effectiveness of such an approach to learn clinical reasoning is lacking [8] and which design features of VPs optimally support the training of clinical reasoning is still not fully understood [9,10].

To address this unresolved issue, we developed a concept mapping tool, which specifically captures the clinical reasoning process while learning with virtual patients and allows a detailed analysis of learners’ reasoning processes [11]. The tool was conceptualized and designed based on a grounded theory exploration of the process of learning clinical reasoning and supports its specific steps [12]. We chose concept mapping as it accounts for the non-linearity of the complex clinical reasoning process and supports the relations of concepts with each other [13].

With this tool, our aim was to analyze use patterns in a real-world educational setting to find out more about learners’ clinical reasoning with virtual patients. Our hypothesis was that there are differences in the clinical reasoning processes between correctly and incorrect diagnosed VPs. Specifically, we wanted to explore the differences in the processes of learners who provided a correct diagnosis on their first attempt and those who required several attempts to reach a correct diagnosis.

## Methods

### Virtual patients and concept mapping tool

We created 67 VPs in German and 30 in English in the VP system CASUS, for a list of VPs see S1 Table.

**Fig 1:**
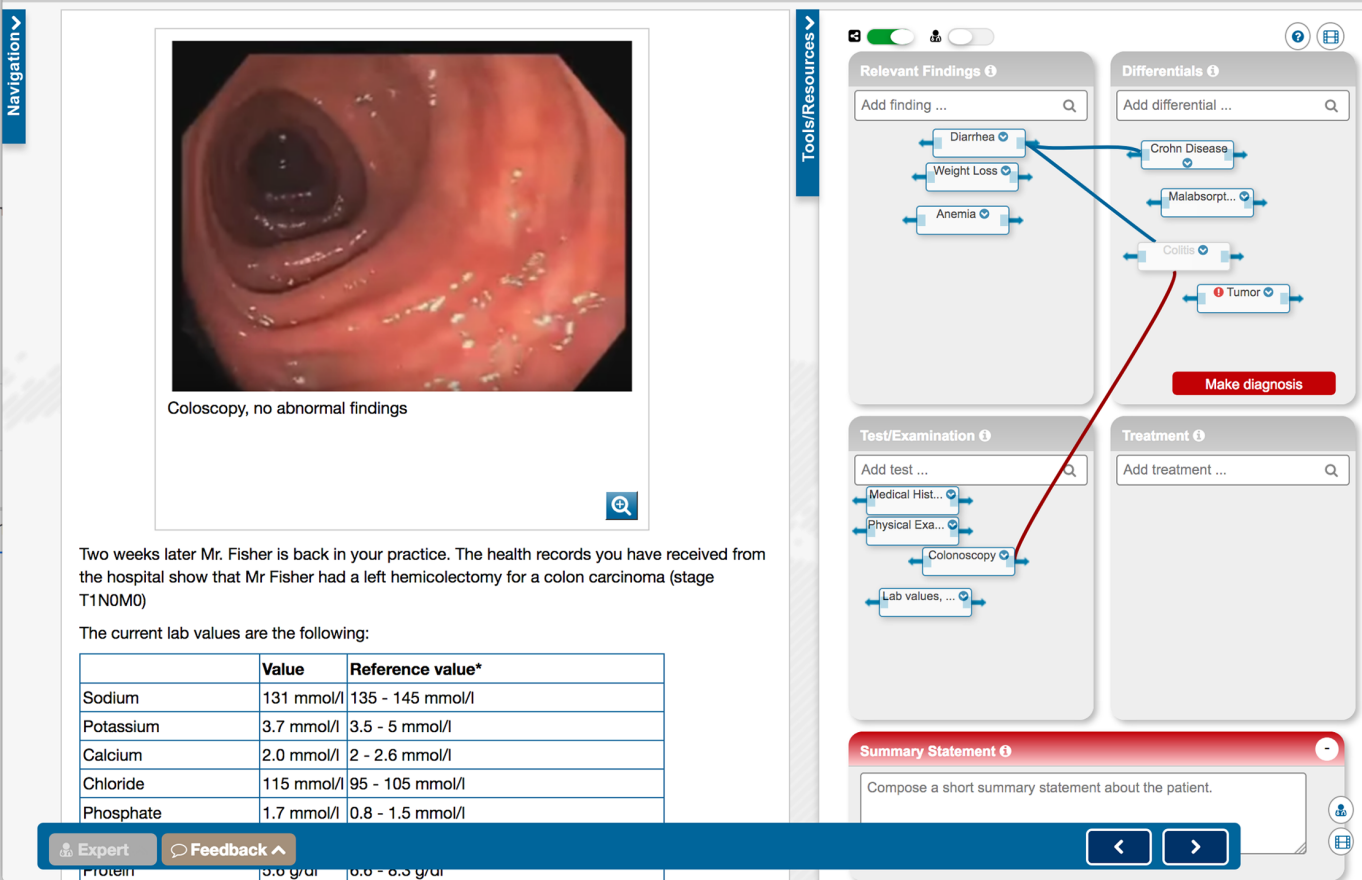
Screenshot of a virtual patient scenario (left side) and the concept mapping tool (right side).

The VPs were combined with a concept mapping tool, which was designed to support the steps of the clinical reasoning process. Learners document their clinical reasoning process by adding elements (also known as "nodes") in four different categories—problems/findings, differential diagnoses they want to consider, tests they would like to perform, such as a physical examination, laboratory tests or medical imaging, and treatment options. Nodes can be connected to indicate relationships, for example a finding confirming a differential diagnosis (Fig 1).

Additionally, learners compose a short summary statement to summarize and prioritize the problems of the patient. Throughout the process, learners may make a final diagnosis and if the diagnosis is incorrect, they may request the correct solution from the system. But, to conclude the scenario, learners must submit a final diagnosis. Errors, such as a premature closure are automatically detected by the system and reported back to the learner.

A physician created the VPs including the expert concept maps covering diseases relevant for medical students from a variety of specialties, such as internal medicine, neurology, or pediatrics. The VPs and maps were reviewed by experts for content accuracy.

Any time during the scenario learners can access an expert map for comparison. Based on this expert map the system automatically scores added nodes and final diagnoses, accounting for synonyms based on a Medical Subject Heading (MeSH) list. The summary statements are scored based on the use of semantic qualifiers [14]. All learners’ interactions with the tool are stored in a relational database. The detailed functionalities, scoring algorithms, and the development process of the tool have been described elsewhere [11]; Table 1 provides an overview of the variables. The selected variables are based on previous work of developing the concept mapping tool [11,12].

**Table 1:** Descriptive statistics and descriptions for variables used in the study.

Variable	Mean	SD	Min	Max	Description
Number of problems	4.94	3.61	0	19	Findings or symptoms the learner identified in a virtual patient
Number of differential diagnoses	4.21	2.72	1	19	Differential diagnoses the learner added to the concept map for each VP.
Number of tests	3.86	3.03	0	17	Tests (e.g. physical exam, laboratory tests, medical imaging) added by the learner.
Number of treatments	1.56	1.81	0	14	Recommended treatments added by the learner
Number of connections	0.76	2.53	0	26	Total connections added by the learner between the nodes in the concept map
Summary statement	0.50	0.50	0	1	Whether learner composed a summary statement summarizing the information about the patient. (yes/no)
Summary statement score	0.31	0.38	0	1	Score of the semantic qualifiers (e.g. "acute" vs. "chronic") in the summary statement, identified from an adapted list provided by Connell et al. [15] Rating algorithm compares counts of the semantic qualifiers used by both the learner to those used by the expert Scored based on an assessment rubric—0 (no use), 0.5 (some use), 1 (concise and complete use) [14].
Confidence	59.45	32.76	0	100	Learner’s confidence with their final diagnosis
Score for problem list	0.14	0.19	0	0.95	Scores of the quality of the problem list, the differential diagnoses, recommended tests and treatments. A heuristic formula was used to calculate the score for each list [11]; an empty list (= no nodes) in a category is scored with zero.
Score for differential diagnosis list	0.11	0.15	0	0.81	
Score for tests list	0.23	0.27	0	0.95	
Score for treatment list	0.11	0.26	0	0.95	
Number of premature closures	0.08	0.27	0	2	Submission of a final diagnosis at an early stage, after which the expert has added finding(s) or tests that are connected to the final diagnosis.
Click on feedback	1.90	3.44	0	28	Number of clicks on the feedback button to consult the expert's map.
Time on task	22.52	22.85	0.6	314	Time in minutes the learner spent on the VP (cumulative from opening or re-opening until closing)

### Participants and data collection

We provided access to two VP courses in German and English to undergraduate medical students. From January 1st until July 31, 2017 access to the courses was free, but registration or login via singleSignOn (Shibboleth, edugain) was required [16].

**Fig 2:**
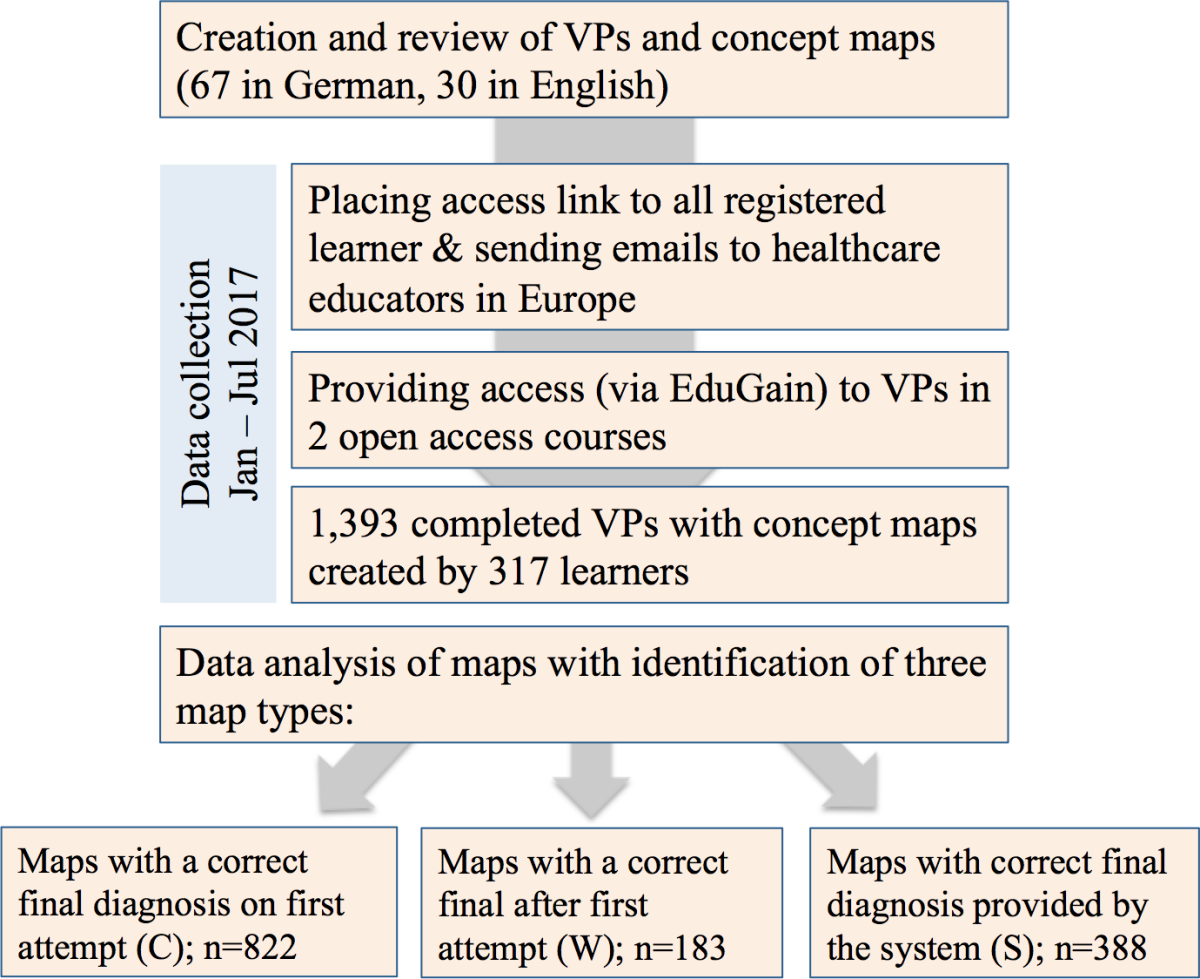
Overview of the study design.

Information about the courses was sent to medical schools in Europe, announced at medical education conferences, and posted on the project's website. Additionally, all registered CASUS users were provided with the link to the new courses in their dashboard. An overview of the study design is shown in Fig 2.

Data collected by the concept mapping tool were anonymous. No personal data, except for an encrypted unique identifier for each user, were transferred from the VP system to the concept mapping tool. If a learner completed a VP multiple times, we only included the first session for our analysis. Anonymized data is published in the Open Science Framework.

### Data analysis

We exported all collected data from the concept mapping tool into Statistical Analysis Software (SAS, SAS Institute Inc. 2013. SAS/STAT 13.1.) for further analysis. Since the focus of this study is the cognitive actions of learners, the unit of analysis was the completed maps (i.e., having a final diagnosis) created by the learners for a VP, rather than the individual learner.

Most of the concept map data are at the time of the first submission of a final diagnosis; the number and scores of treatments, time on task and feedback clicks were analyzed at the end of a scenario.

We examined average differences in scores and use patterns using linear mixed modeling (LLM) and multinomial logistic regression using generalized estimating equations (GEE) [17] to account for the correlated errors associated with the nested structure of the data. We used correlations (pearson-product moment and point-biserial) to examine the patterns of associations between the number of nodes and scores and present these results as a heat map to focus on the broad patterns. Basic data on the learners are recorded in the VP system CASUS upon registration. But, there is no transfer of any personal data to the concept mapping tool.

### Ethical approval

We obtained ethical approval from the ethical committee at Ludwig-Maximilians Universität Munich, Germany (reference number: 260–15).

## Results

### Learner demographics

Overall, 858 undergraduate medical students enrolled in the two courses during the study period (139 in English, 718 in German); 317 users (36.5%) completed at least one virtual patient with a final diagnosis. From these 317 users 87 were male (27.4%), 168 female (53.0%) (62 missing values).

### Completed maps

Overall, we recorded 1,393 completed concept maps during the study time, which were created by 317 different users, from which 47.6% (n = 151) completed one map, 13.9% (n = 44) completed two maps, and 38.5% (n = 122) completed three or more concept maps. We found that in 59.0% (n = 822) of the maps the correct final diagnosis was provided on the first attempt (Group C). For the maps that were not solved correctly on the first attempt, the correct final diagnosis was made after multiple attempts in 13.1% (n = 183) of the maps (Group W), and in 27.9% (n = 388) of the maps learners gave up and requested the correct solution from the system (Group S).

In group S, in 59.5% (n = 231) of the maps learners gave up after the first attempt and another 25.3% (n = 98) after the second attempt; the maximum number of attempts was 17. In group W in 66.7% (n = 122) of the maps, learners submitted the correct final diagnosis on the second attempt, and 15.9% (n = 29) on the third attempt. Maximum number of attempts was seven.

38% (n = 122) of the learners submitted three or more maps belonging to more than one group. Of these learners, we found that only 7.4% (n = 9) created maps that belonged solely in one of the three groups (e.g., all maps in C, W, or S). Most created maps that belonged in two or three groups (45.9%, n = 56 and 46.7%, n = 57, respectively).

### Use patterns and scores

**Fig 3:**
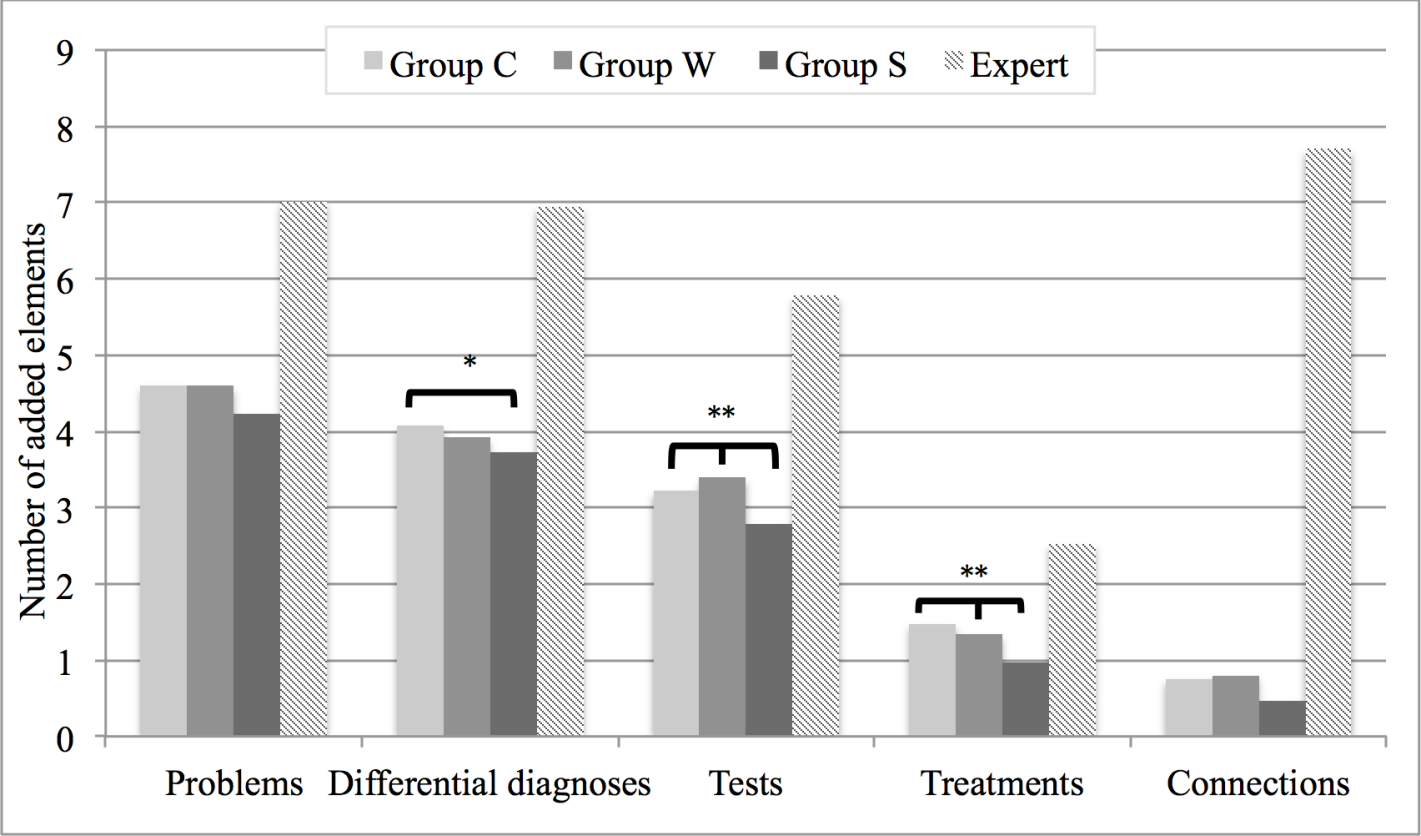
Average number of elements—added nodes in each category and number of added connections—for the three groups and the expert maps. *significant difference between group C (correct diagnosis was made on first attempt) and S (correct diagnosis provided by the system) (p<0.05), ** significant difference between group S and groups C and W (correct final diagnosis was submitted after first attempt).

For the three groups of maps, we saw differences in the use patterns (i.e, number of nodes and connections) and the scores earned for the specific clinical reasoning activities. In group S, the maps contained fewer problems, differential diagnoses, tests, treatment options, and connections than in groups C and W. Differences between group C and W were not significant. For all three groups, the average number of connections was low compared to the expert maps (Fig 3).

When looking into the details of the map development, maps in group S had significantly fewer summary statements, were scored lower in all categories, and learners in this group were less confident with their final diagnosis decision. Also, the expert map was consulted less frequently and learners spent less time on creating the maps (Table 2). The only significant difference between the groups C and W, was a lower score for the differential diagnoses in group W.

**Table 2:** Average scores, confidence with final diagnosis, time on task, and feedback requests by groups of concept maps—Group C (correct diagnosis was made on first attempt), group W (correct final diagnosis was submitted after first attempt) and group S (correct diagnosis provided by the system).

Variable	Group C	Group W	Group S
Number of maps	822 (59.0%)	183 (13.1%)	388 (27.9%)
Summary statement composed*	57% ^a^ (n = 458)	58% ^b^ (n = 105)	38% ^ab^ (n = 128)
Summary statement score	0.25 ^a^	0.21	0.18 ^a^
Mean confidence	63.95% ^a^	62.64% ^b^	50.26% ^ab^
Score for problem list	0.15 ^a^	0.14 ^b^	0.09 ^ab^
Score for differential list	0.14 ^ac^	0.10 ^bc^	0.04 ^ab^
Score for test list	0.21 ^a^	0.24 ^b^	0.18 ^ab^
Score for treatment list	0.10	0.12 ^a^	0.07 ^a^
Premature closure*	-	0.18 ^a^	0.09 ^a^
Click on feedback	1.98	2.19	1.87
Time on task	20.57 min	20.83 min	18.91 min

Averages in each row with the same superscript letters differ significantly (with Tukey HSD, p< .05, at least).

*Generalized Estimating Equations (GEE); other analyses are generalized linear mixed modeling

### Correlations

**Fig 4:**
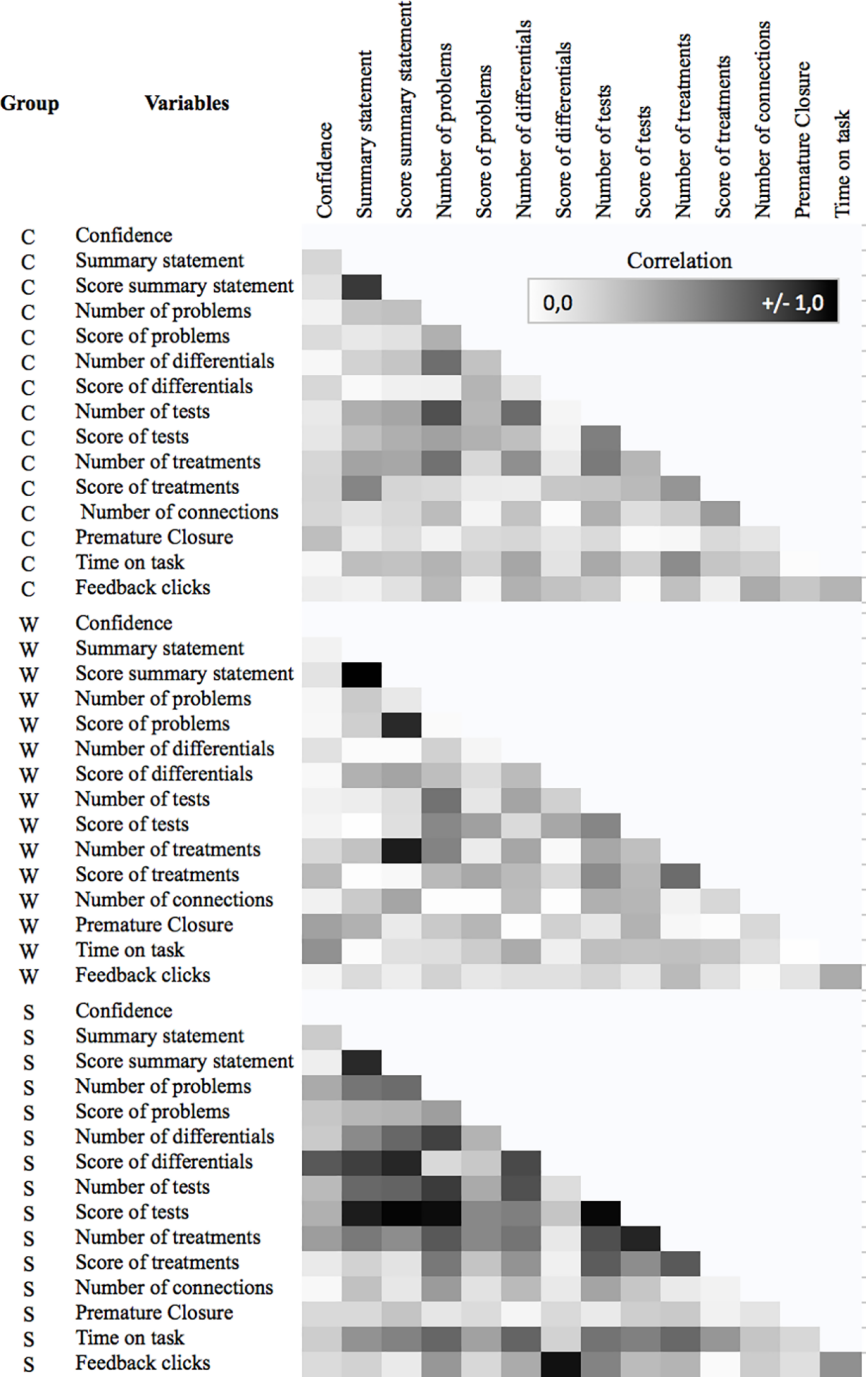
Correlations between variables in the three groups Group C (correct diagnosis was made on first attempt), group W (correct final diagnosis was submitted after first attempt) and group S (correct final diagnosis provided by the system).

The correlations between the number of added nodes and scores in the four categories (problems, differential diagnoses, tests, treatments) were higher in group S than in groups C and W (Fig 4, S2 Table). For example, the correlation between the number of recommended tests and quality of the test (measured by scores) was quite high in group S (r = .97), and much lower in groups C and W (r = .50 and .48, respectively). Also, compared to groups W and C, the presence of a summary statement was related to the higher scores in group S for the differential diagnosis (r = .75), tests (r = .89), and had a moderate correlation with the numbers of problems, tests, treatment options, and differentials. We also detected a large difference in correlations between the groups for the number of clicks on the expert map as feedback.

### Multinomial logistic regression

We used a multinomial logistic regression to understand the combined differences in use patterns and scores between the three groups (S1 Table). Group W and S were compared to the reference group C. Controlling for other variables in the model, a premature closure was more likely to occur in group W than group S. Recommending more tests was significantly more likely in group W, compared to group C. Lower numbers of feedback clicks, suggesting fewer treatment options, and a lower confidence in their final diagnoses was more prominent in group S, than in group C. Lower scores on the differential diagnoses and problem lists were more probable for groups W and S. Compared to group C, higher scores on tests occurred more with group W and lower summary statement scores were more evident in group S.

## Discussion

The results of our study partially confirm our hypothesis that there is a significant difference in the clinical reasoning processes for learners. However, the relevant determinant is not the correct solution on the first attempt or subsequent attempts, but whether the correct final diagnosis was made by the learners themselves (groups C or W) or whether the solution was requested from the system (group S). In the following we will discuss the results in more detail.

Overall, the differences between the maps in groups C and W were small and non-significant, whereas the maps in group S contained significantly fewer nodes and lower scores in all four categories (problems, differential diagnoses, tests, and treatment options) as well as fewer connections compared to group C and W. A potential explanation could be that for some learners these VPs were more difficult, leading them to give up on finding the correct final diagnosis. However, learners spent less time on these VPs and requested feedback from the expert less often than what we would have expected with more difficult VP scenarios. Another explanation could be that for sessions in group S, learners might have been less motivated and engaged. However, the results show that the maps of most learners were at least associated with two groups, suggesting that learners were generally motivated to work with the VPs. Further research is needed to investigate the VP characteristics in the three groups and better understand the reasons and the role of feedback in the clinical reasoning process.

Compared to the number of connections drawn by the expert, the maps in all three groups included a very low number of connections (Fig 3). We can only hypothesize about the reasons, which might be a usability issue, a need for more instruction about the importance of connections in concept maps, or challenges faced by the learners in reflecting why and how the nodes of their map are connected.

Overall, the scores in all categories were quite low, because those nodes where the learner has already seen the expert map are scored as a zero.

If learners gave up on providing a final diagnosis (group S), a summary statement was composed significantly less often, and if it was composed, it was scored significantly lower based on the use of semantic qualifiers than the summary statements in groups C and W. Research has shown that composing a summary statement in both face-to-face teaching and virtual scenarios allows learners to organize and present relevant aspects, and to practice using semantic qualifiers [18,19], which are related to diagnostic accuracy [20,21]. Our study extends these findings by showing that for group S, composing a summary statement or a summary statement with adequate use of semantic qualifiers is related to more nodes in all four categories and higher scores on differential diagnoses and tests.

Interestingly for group C, the relationship between a summary statement composition and the score for treatments is lower, and for group W we find a high correlation between the quality of the summary statement and the score for the problem list and the number of added treatments. Thus, we can assume that the careful composition of a summary statement might be more beneficial for learners when they are struggling with structuring their thoughts and determining the correct final diagnosis.

A premature closure error occurred significantly more often in group W, than in group S. At the same time group W was slightly less confident than group C and significantly more confident than group S. This finding adds quantitative data to a recent mixed-method study, indicating that a variety of errors are made by medical students during their reasoning process [22]. Friedman et al. showed that final year medical students were less accurate and less confident in their diagnostic decisions compared to attending physicians [23]. Our study further indicates that within the group of medical students there are significant differences in the level of confidence for VPs. This finding warrants further exploration about the reasons for overconfidence, including attitudinal and cognitive factors [24]. Additionally, we have an excellent opportunity to provide detailed feedback to learners to help them learn from errors and overconfidence in a safe environment, and to address the lack of a formal cognitive error and patient safety curriculum [25].

We are aware that our study has some limitations. First, due to the anonymous data collection we do not have any information on the learners who completed the VP scenarios. Thus, we cannot take into account any contextual and person-specific factors, such as motivation, level of expertise, or demographic data.

Second, the data collection was intentionally not conducted in a controlled setting, but, using an approach, which is comparable to big data studies. The focus of big data studies is on studying user behavior and usage patterns, thus we believe it is an appropriate method for avoiding biases often involved in artificial controlled study settings, such as motivation or selection. Third, we carefully tracked all user actions with timestamps and did not detect any signs for technical problems that could cause a learner to spend exceptionally more time on a VP. We also did not receive any support requests or complaints regarding technical problems. Nevertheless we cannot rule out that on rare occasions the time on task might have been prolonged due to technical issues.

## Conclusions

Overall, our results indicate that diagnostic accuracy in the form of correctness of the final diagnosis in the first attempt should be reconsidered as a sole indicator of clinical reasoning competence. In our study, the greatest difference in the clinical reasoning process was between those learners who were able to identify a correct final diagnosis—no matter how many attempts it took versus those who gave up and requested the solution from the system.

"One shot" approaches focusing on the first attempt to provide a final diagnosis, are not patient-centered or realistic, even if they are widely used in VPs, clinical reasoning research studies, and training in general. In reality, a healthcare professional would not stop diagnostics if their first diagnosis turned out to be incorrect. Thus, for the training, assessment, and research of clinical reasoning we suggest focusing more on the details of the process to reach a correct diagnosis, rather than whether it was made in the first attempt. In VP scenarios, learners often have to make a decision about the final diagnosis without having the opportunity to retry or request the solution from the system. Consequently, it has not been possible to make the important distinction between the learners giving up and those reaching the correct final diagnosis by revising their diagnoses.

### Outlook

Our study successfully measures and visualizes the clinical reasoning process and the development of a final diagnosis. Furthermore, the use of concept mapping is an innovative approach to measuring the iterative and non-linear thought processes inherent in clinical reasoning [13].

Based on the results of this study we will continue to develop the concept mapping tool including more dynamic scaffolding and feedback elements to specifically support learners who have problems composing a summary statement and struggle to submit the correct final diagnosis. We concur with Berman et al. that VPs can be used for research that will improve medical curricula [26]. To this end, our approach of combining VPs with a structured clinical reasoning tool raises some important questions about clinical reasoning instruction, which should be investigated further.

To date, the VP courses have not been formally integrated into a curriculum. Thus, we intend to expand the courses and integrate them into healthcare curricula, especially longitudinal courses dedicated to clinical reasoning training and adopting a “mixed practice” of topics and specialties [2]. However, this may be challenging since often there is no structured clinical reasoning curriculum. This gap in instructional practice [8] may be a place where VPs and the concept mapping tool could be a valuable component.

## Supporting information

S1 Table List of virtual patients used for our study. (DOCX)

S2 Table Multinomial logistic regression for the W (Wrong) and S (System Solution) groups compared to the C (Correct) group. (DOCX)
