# Supplementary material for: Uncovering the relation between clinical reasoning and diagnostic accuracy – An analysis of learner's clinical reasoning processes in virtual patients
Source: PLoS One. 2018 Oct 4;13(10):e0204900. doi: 10.1371/journal.pone.0204900 (PMC6171878; doi:10.1371/journal.pone.0204900)
Supplement: S1 Table — (DOCX) [file pone.0204900.s001.docx]

Appendix 1: List of virtual patients (VP)

| **No** | **Name of VP** | **Key finding** | **Final diagnosis** | **Number of completed maps** | **Language**  (de=German en=English) |
| --- | --- | --- | --- | --- | --- |
| 1 | Ahmed Ünal | Abdominal pain | Hernia | 79 | de |
| 2 | Alan Britten | Cough | Bronchial carcinoma | 4 | de |
| 3 | Alexandra May | Abdominal pain | Appendicitis | 20 | de, en |
| 4 | Anja Winterberg | Tachycardia | Hyperthyreoidosis | 1 | de |
| 5 | Anna-Lena Schmidt | Headache | Evans syndrome | 1 | de |
| 6 | Annemarie Winkel | Hypertension | Polycytemia vera | 2 | de |
| 7 | Bernhard Hinkel | Pain | Humerus fracture | 2 | de |
| 8 | Britta Ohland | Epistaxis | Hypertension | 38 | de, en |
| 9 | Calvin Warner | Dyspnea | COPD exacerbation | 33 | de, en |
| 10 | Carl Berner | Hematemesis | Mallory-Weiss syndrome | 41 | de, en |
| 11 | Carl Zimmerman | Chest pain | Myocardial infarction | 62 | de, en |
| 12 | Carola Kaufmann | Pain | Gonarthrosis | 14 | de |
| 13 | Carolina Bach | Dyspnea | Pneumonia | 35 | de, en |
| 14 | Clark Wilman | Abdominal pain | Functional dyspepsia | 3 | de, en |
| 15 | Dominik Maller | Fever | Myocarditis | 3 | de |
| 16 | Elena von Dewitz | Pain | Femur fracture | 9 | de |
| 17 | Emma Kruger | Dyspnea | Pulmonary embolism | 60 | de, en |
| 18 | Erik Marte | Headache | Subarachnoidal Bleeding | 9 | de |
| 19 | Eva Miller | Diarrhea | Colitis ulcerosa | 59 | de, en |
| 20 | Felix Meindl | Headache | Macroprolactinoma | 5 | de |
| 21 | Franziska Rosen | Dyspnea | Systemic sclerosis | 24 | de |
| 22 | Fred Bower | Diarrhea | Pseudomembraneous colitis | 34 | de, en |
| 23 | Gabriele Krause | Abdominal pain | Cholecystitis | 19 | de |
| 24 | Gerald Fuchs | Vomiting | Glomerulonephritis | 2 | de |
| 25 | Heidi Wagner | Recurring infections | Thyreoid carcinoma | 2 | de |
| 26 | Robert Baley | Fever | Pneumonia | 137 | de, en |
| 27 | Herbert Mittermaier | Hypertension | Apnoe syndrome | 1 | de |
| 28 | Isabel Schuster | Dyspnea | Asthma | 9 | de |
| 29 | James King | Diarrhea | Ascariasis | 2 | de, en |
| 30 | Jan Fisher | Diarrhea | Celiac disease | 29 | de |
| 31 | Jelena Jakovic | Headache | Cluster headache | 9 | de |
| 32 | Johannes Maier | Dyspnea | Pneumothorax | 25 | de, en |
| 33 | Josef Schröder | Dyspnea | Pleural effusion | 1 | de |
| 34 | Julia Biederman | Abdominal pain | Ulcus duodeni | 7 | de, en |
| 35 | Kai Thälmann | Abdominal pain | Urolithiasis | 6 | de |
| 36 | Karim Murasic | Chest pain | Aortic valve stenosis | 32 | de, en |
| 37 | Karina Schmitt | Fatigue | Autoimmune hepatitis | 17 | de |
| 38 | Katharina Rigger | Headache | Migraine | 1 | de |
| 39 | Krystyna Kowalczyk | Splenomegaly | Leukemia (CML) | 7 | de, en |
| 40 | Kurt Baier | Tachycardia | Hyperthyreoidosis | 4 | de |
| 41 | Leslie Smith | Abdominal pain | Chronic pancreatitis | 3 | de, en |
| 42 | Lilly Coster | Syncope | ARDS | 41 | de, en |
| 43 | Ludwig Spänle | Fatigue | Leukemia (CLL) | 2 | de |
| 44 | Maria Bauer | Syncope | Colon carcinoma | 13 | de, en |
| 45 | Marlene Reister | Pain | Rheumatoid arthritis | 17 | de |
| 46 | Martha Eberle | Hematoma | Hemophilia A | 6 | de |
| 47 | Martin Spelsberg | Fatigue | Hepatitis E | 8 | de, en |
| 48 | Martina Fielding | Diarrhea | Giardiasis | 12 | de, en |
| 49 | Melanie Weber | Fever | Mononucleosis | 77 | de, en |
| 50 | Miko Sasagawa | Vomiting | Type-I diabetes | 2 | de |
| 51 | Miroslav Jasic | Cough | Asbestosis | 6 | de, en |
| 52 | Norman Jacobs | Abdominal pain | Hepatitis C | 1 | de |
| 53 | Patrick Geller | Hematemesis | Liver cirrhosis | 31 | de, en |
| 54 | Pia Horcek | Arrhytmia | Atrial fibrillation | 4 | de |
| 55 | Ralf Braun | Pain | Weber-B fracture | 1 | de |
| 56 | Reinhard Bachmeier | Headache | Adenoma hypophysis | 3 | de |
| 57 | Robert Wallner | Syncope | WPW syndrome | 45 | de, en |
| 58 | Sarah Carter | Fever | Malaria | 40 | de, en |
| 59 | Stefan Müller | Fatigue | Type-I diabetes | 9 | de |
| 60 | Stephanie Turner | Cough | Asthma | 38 | de, en |
| 61 | Sybille Jonas | Syncope | Bronchial carcinoma | 47 | de, en |
| 62 | Thomas Sachs | Dyspnea | Renal failure | 45 | de, en |
| 63 | Ulrike Birnbaum | Chest pain | Broken heart syndrome | 8 | de |
| 64 | Veronika Heidemann | Dysuria | Pyelonephritis | 11 | de |
| 65 | Walter Mertens | Cough | Tuberculosis | 38 | de, en |
| 66 | Werner Schuster | Vomiting | Duodenal stenosis | 20 | de |
| 67 | Yara Mahmoudi | Fever | Meningitis | 17 | de |
