# Supplementary material for: Uncovering the relation between clinical reasoning and diagnostic accuracy – An analysis of learner's clinical reasoning processes in virtual patients
Source: PLoS One. 2018 Oct 4;13(10):e0204900. doi: 10.1371/journal.pone.0204900 (PMC6171878; doi:10.1371/journal.pone.0204900)
Supplement: S2 Table — (DOCX) [file pone.0204900.s002.docx]

Appendix 2 Multinomial logistic regression for the W (Wrong) and S (System Solution) groups compared to the C (Correct) group.

Panel A. Use Patterns

|  | **Group W** | | | | | **Group S** | | | | |
| --- | --- | --- | --- | --- | --- | --- | --- | --- | --- | --- |
|  | **Coefficient (SE)** | | **Odds Ratio** | **95% CI** | | **Coefficient (SE)** | | **Odds Ratio** | **95% CI** | |
| Intercept | -1.910^c^ | (0.29) |  | -2.471, | -1.348 | 0.730^c^ | (0.19) |  | 0.355, | 1.105 |
| Time on Task (in Min) | -0.004 | (0.00) | 1.00 | -0.013, | 0.005 | 0.003 | (0.00) | 1.00 | -0.004, | 0.010 |
| Confidence in final diagnosis | -0.004 | (0.00) | 1.00 | -0.010, | 0.002 | -0.015^c^ | (0.00) | 0.99 | -0.019, | -0.010 |
| Clicks on feedback | 0.005 | (0.03) | 1.01 | -0.047, | 0.057 | -0.051^z^ | (0.03) | 0.95 | -0.103, | 0.000 |
| Premature closure | 1.493^c^ | (0.27) | 4.45 | 0.971, | 2.015 | 0.607^a^ | (0.27) | 1.84 | 0.085, | 1.130 |
| Differential diagnoses | -0.033 | (0.04) | 0.97 | -0.111, | 0.046 | -0.047 | (0.03) | 0.95 | -0.116, | 0.021 |
| Problems | 0.037 | (0.03) | 1.04 | -0.023, | 0.097 | 0.026 | (0.03) | 1.03 | -0.025, | 0.077 |
| Tests | 0.105^b^ | (0.04) | 1.11 | 0.026, | 0.184 | -0.036 | (0.04) | 0.96 | -0.109, | 0.036 |
| Treatments | 0.005 | (0.05) | 1.01 | -0.101, | 0.111 | -0.199^c^ | (0.06) | 0.82 | -0.311, | -0.087 |
| Connections | -0.005 | (0.03) | 0.99 | -0.069, | 0.059 | -0.029 | (0.04) | 0.97 | -0.100, | 0.041 |
| Summary statement submitted | -0.074 | (0.22) | 0.93 | -0.505, | 0.357 | -0.413^a^ | (0.17) | 0.66 | -0.754, | -0.072 |

Note: a=p< .05, b=p < .01, c=p< .001, z=p< .10

Panel B. Scores

|  | **Group W** | | | | | **Group S** | | | | |
| --- | --- | --- | --- | --- | --- | --- | --- | --- | --- | --- |
| **Effect** | **Coefficient (SE)** | | **Odds Ratio** | **95% CI** | | **Coefficient (SE)** | | **Odds Ratio** | **95% CI** | |
| Intercept | -1.372^c^ | (0.24) |  | -1.850, | -0.893 | 1.018^c^ | (0.19) |  | 0.635, | 1.400 |
| Time on Task (in Min) | -0.001 | (0.00) | 1.00 | -0.009, | 0.007 | -0.003 | (0.00) | 1.00 | -0.011, | 0.005 |
| Confidence in final diagnosis | -0.002 | (0.00) | 1.00 | -0.008, | 0.004 | -0.013^c^ | (0.00) | 0.99 | -0.018, | -0.008 |
| Score for differential diagnoses list | -2.477^c^ | (0.66) | 0.08 | -3.769, | -1.185 | -7.388^c^ | (0.85) | 0.00 | -9.059, | -5.717 |
| Score for problem list | -0.233 | (0.47) | 0.79 | -1.149, | 0.682 | -1.021^a^ | (0.47) | 0.36 | -1.946, | -0.097 |
| Score for treatments | 0.462 | (0.31) | 1.59 | -0.141, | 1.064 | 0.201 | (0.33) | 1.22 | -0.439, | 0.841 |
| Score for tests | 1.096^b^ | (0.34) | 2.99 | 0.424, | 1.767 | 0.032 | (0.34) | 1.03 | -0.632, | 0.696 |
| Summary Statement Score | -0.431 | (0.27) | 0.65 | -0.968, | 0.105 | -1.011^c^ | (0.26) | 0.36 | -1.516, | -0.506 |

Note: a=p< .05, b=p < .01, c=p< .001, z=p< .10
